# New approaches to measuring anthelminthic drug efficacy: parasitological responses of childhood schistosome infections to treatment with praziquantel

**DOI:** 10.1186/s13071-016-1312-0

**Published:** 2016-01-27

**Authors:** Martin Walker, Tarub S. Mabud, Piero L. Olliaro, Jean T. Coulibaly, Charles H. King, Giovanna Raso, Alexandra U. Scherrer, J. Russell Stothard, José Carlos Sousa-Figueiredo, Katarina Stete, Jürg Utzinger, Maria-Gloria Basáñez

**Affiliations:** Department of Infectious Disease Epidemiology, School of Public Health, Faculty of Medicine (St Mary’s Campus), Imperial College London, London, W2 1PG UK; London Centre for Neglected Tropical Disease Research, Department of Infectious Disease Epidemiology, Imperial College London, London, W2 1PG UK; UNICEF/UNDP/World Bank/WHO Special Programme on Research and Training in Tropical Diseases (TDR), World Health Organization, Av. Appia 20, CH-1211, Geneva 27, Switzerland; Centre for Tropical Medicine and Global Health Nuffield Department of Medicine Research Building, University of Oxford Old Road Campus, Roosevelt Drive, Oxford, OX3 7FZ UK; Department of Epidemiology and Public Health, Swiss Tropical and Public Health Institute, P.O. Box, CH-4002, Basel, Switzerland; University of Basel, P.O. Box, CH-4003, Basel, Switzerland; Unité de Formation et de Recherche Biosciences, Université Félix Houphouët-Boigny, 22 BP 770, Abidjan 22, Côte d’Ivoire; Centre Suisse de Recherches Scientifiques en Côte d’Ivoire, 01 BP 1303, Abidjan 01, Côte d’Ivoire; Center for Global Health and Diseases, Case Western Reserve University, 2109 Adelbert Road, Cleveland, OH 44106 USA; Schistosomiasis Consortium for Operational Research and Evaluation, University of Georgia, 500 D W Brooks Drive, Athens, GA 30602 USA; Division of Infectious Diseases and Hospital Epidemiology, University Hospital Zurich, University of Zurich, Rämistrasse 100, CH-8091 Zurich, Switzerland; Department of Parasitology, London School of Tropical Medicine, Liverpool, L3 5QA UK; Centro de Investigação em Saúde de Angola (Health Research Center in Angola), Rua direita do Caxito, Hospital Provincial, Bengo, Angola; Department of Life Sciences, Natural History Museum, Wolfson Wellcome Biomedical Laboratories, Cromwell Road, London, SW7 5BD UK; Center for Infectious Diseases and Travel Medicine, Department of Medicine, University Hospital Freiburg, Hugstetter Strasse 55, D-79106 Freiburg, Germany; Present address: Stanford School of Medicine, 291 Campus Drive, Stanford, CA 94305 USA

**Keywords:** Schistosomiasis, Praziquantel, Anthelminthic drug efficacy, Egg reduction rate, Conditional models, Marginal models, Bayesian methods

## Abstract

**Background:**

By 2020, the global health community aims to control and eliminate human helminthiases, including schistosomiasis in selected African countries, principally by preventive chemotherapy (PCT) through mass drug administration (MDA) of anthelminthics. Quantitative monitoring of anthelminthic responses is crucial for promptly detecting changes in efficacy, potentially indicative of emerging drug resistance. Statistical models offer a powerful means to delineate and compare efficacy among individuals, among groups of individuals and among populations.

**Methods:**

We illustrate a variety of statistical frameworks that offer different levels of inference by analysing data from nine previous studies on egg counts collected from African children before and after administration of praziquantel.

**Results:**

We quantify responses to praziquantel as egg reduction rates (ERRs), using different frameworks to estimate ERRs among population strata, as *average* responses, and within strata, as *individual* responses. We compare our model-based average ERRs to corresponding model-free estimates, using as reference the World Health Organization (WHO) 90 % threshold of optimal efficacy. We estimate distributions of individual responses and summarize the variation among these responses as the fraction of ERRs falling below the WHO threshold.

**Conclusions:**

Generic models for evaluating responses to anthelminthics deepen our understanding of variation among populations, sub-populations and individuals. We discuss the future application of statistical modelling approaches for monitoring and evaluation of PCT programmes targeting human helminthiases in the context of the WHO 2020 control and elimination goals.

**Electronic supplementary material:**

The online version of this article (doi:10.1186/s13071-016-1312-0) contains supplementary material, which is available to authorized users.

## Background

Human helminthiases comprise six of the seven most prevalent neglected tropical diseases (NTDs) [1] that are targeted by the World Health Organization (WHO) for elimination, where feasible, by 2020 [2]. Schistosomiasis infects over 250 million people with an estimated global burden of 3.31 million disability-adjusted life years [3, 4]. The main strategy to control and eliminate human helminthiases is preventative chemotherapy (PCT) by mass drug administration (MDA) using a handful of safe and efficacious anthelminthic drugs [5–7]. The cornerstone of schistosomiasis control and elimination efforts is praziquantel which is effective in killing adult *Schistosoma mansoni*, *S. haematobium* and *S. japonicum,* the most globally important causes of intestinal, urogenital and Asian intestinal schistosomiasis, respectively.

The reliance on single drugs with essentially no available alternatives makes the long-term effectiveness of the PCT strategy susceptible to the potentially devastating consequences of emerging anthelminthic resistance. Although examples of praziquantel-resistant isolates of *S. mansoni* are currently scant and virtually non-existent for *S. haematobium* or *S. japonicum* [8], there is broad consensus that the efficacy of praziquantel—and other anthelminthics used for MDA—should be monitored to detect atypical responses that may indicate dwindling efficacy, possibly caused by emerging drug resistance [6, 9–12].

The efficacy of anthelminthics is typically expressed as either a cure rate (CR), or an intensity reduction rate (IRR; Table 1), calculated using data on parasite transmission stages collected before and after treatment. Intensity reduction rates are recommended by the WHO for monitoring the efficacy of both praziquantel and the benzimidazoles, which are widely used in PCT targeting soil-transmitted helminthiasis [13]. In this context, IRRs are calculated using data on helminth egg counts and so are referred to as egg reduction rates (ERRs). The methods of estimating ERRs can be divided into model-free and model-based approaches.

**Table 1:** Definitions

Term	Definition
Bayesian credible interval (BCI)	An interval of a posterior distribution that defines the domain within which the value of a parameter lies with a specified probability (typically 0.95 or 95 %). The Bayesian analogy to the classical frequentist confidence interval.
Best linear unbiased prediction (BLUP)	A frequentist technique used in linear mixed models to estimate random effects terms, so-called empirical best linear unbiased predictors (EBLUPs).
Bootstrapping	A numerical resampling technique typically used to generate estimates of uncertainty associated with calculated statistical quantities.
Cure rate (CR)	Proportion of individual hosts positive for parasites who become parasitologically negative after treatment.
Intensity reduction rate (IRR)/egg reduction rate (ERR)	The intensity of infection after treatment expressed as a proportion of the intensity of infection before treatment. For schistosomiasis (and soil-transmitted helminthiases), this is typically expressed as an egg reduction rate; the egg count after treatment expressed as a proportion of the egg count before treatment.
Drug response	Dynamics of parasite (transmission) stages following anthelminthic treatment.
Fixed effect	The component of an effect exerted by a particular value or level of a covariate that is the same among all observations within a unit of a structured dataset
Generalized estimating equation (GEE)	A technique for estimating the parameters of a marginal model fitted to correlated repeated measures (observations). The GEE approach is semi-parametric because it relies on the first two moments of the observed data, but not on the full likelihood.
Generalized linear model (GLM)	An extension of the simple linear regression model that is compatible with error distributions from any of the exponential family of probability distributions, including the normal, Poisson, binomial, and gamma distributions. The simple linear regression model is a GLM with normally distributed errors.
Conditional (linear) mixed model (also called a generalized linear mixed model, GLMM)	An extended GLM that includes a linear predictor comprised of covariate coefficients that exert both fixed and random effects.
Hyperparameter	A parameter in a hieracrchical or multilevel statistical model that governs the distribution of lower-level random effects terms
Marginal model	An adaptation of a GLM for use with correlated repeated measures (observations). Marginal refers to the marginal mean of observations from individuals (units) sharing a set of covariates. A marginal model comprises three model components; a marginal mean, which depends on covariates; a marginal variance, which is typically a function of the marginal mean, and a correlation structure for the repeated measures.
Markov chain Monte Carlo (MCMC)	A stochastic algorithm central to Bayesian statistical inference which samples parameter values from the posterior probability distribution by combining information from the likelihood of the observed data and the prior probability distribution of the parameters.
Random effects	The component of an effect exerted by a particular value or level of a covariate that is different among observations within a unit of a structured dataset. The magnitude of the deviations from the fixed effect component is governed by (typically a normal) distribution defined by estimable hyperparameters.
Repeated measures	Measurements or observations made repeatedly on the same unit, for example, multiple schistosome egg counts measured from the same individual host.
Restricted maximum likelihood (REML) estimation	An alternative to maximum likelihood (ML) estimation for models that include random effects. In REML estimation, the dispersion of the random effects is estimated having averaged over some of the uncertainty in the fixed effects. By contrast, in ML estimation, the fixed effects estimates are treated as precisely correct.
Sandwich estimator	A standard error (SE) of an estimated quantity that is robust to misspecifications in the variance-covariance of the error distribution in a statistical model. Sandwich estimators are typically used with marginal models so that SEs (and confidence intervals) are invariant to inaccuracies in the specification of the repeated measures correlation structure. In this context, sandwich estimators are based on the empirically observed variation among unit-level statistics rather than on the model-derived variance-covariance matrix which depends on the assumed correlation structure.

Definitions are taken from Walker *et al*. [16]

Model-free approaches calculate ERRs directly from data using simple arithmetic operations, without invocation of distributional (modelling) assumptions. These so-called sample estimates are easy to calculate and straightforward to interpret as population averages [14] and are the most commonly reported estimates of efficacy [15, 16]. Although egg count values are not normally distributed even after log transformation, the WHO recommends arithmetic means over geometric means [13] as the former are more sensitive to outliers and thus more apt to identify suboptimal group responses [14]. However, they are not readily compatible with exploring associations between efficacy and covariates, nor do they permit inference on the underlying distribution of drug responses among individuals.

Model-based approaches are seldom used to estimate efficacy, despite offering a powerful means to conduct multivariate analyses of longitudinal data [17] on egg counts to delineate and compare efficacy among individuals, among groups of individuals and among populations. Furthermore, modelling approaches, particularly in conjunction with Bayesian techniques, can be used to define distributions of responses to anthelminthics among individuals within demographic strata and among populations [16].

Here, we illustrate two distinct modelling approaches by analysing data on schistosome egg counts collected from children infected with *S. mansoni* or *S. haematobium* before and up to six weeks after administration of praziquantel. The data are from several past studies on the efficacy of praziquantel from communities in Côte d’Ivoire, Kenya and Uganda, predominantly naïve to MDA, or having received only a few rounds of MDA. We illustrate how two classes of statistical model can be used to (i) identify geographical, demographic and drug regimen covariates associated with ERRs and (ii) explore distributions of individual responses to praziquantel in key population demographics and evaluate the frequency of nominally optimal and sub-optimal responders. We discuss the context in which each modelling approach is most appropriate, depending on the goal of the analysis and the level of inference sought [18]. We also describe how distributions of drug responses among individuals infected with predominantly drug-naïve and maximally susceptible parasites could facilitate identification of sub-optimally or atypically responding individuals, ultimately providing a practical tool for the monitoring and evaluation (M&E) of anthelmintic efficacy during PCT programmes.

## Models and methods

### Ethics, consent and permissions

This paper reports a secondary analysis of data collected in past studies, all of which obtained the necessary ethical approvals from relevant institutional review boards and local and national ethics committees. All data were collected in accordance with international ethical standards. Data received were completely and irreversibly anonymised. Additional information can be found in the original publications and in Additional file 1: Supplementary Tables, Table S1.

### Data selection criteria

**Fig. 1:**
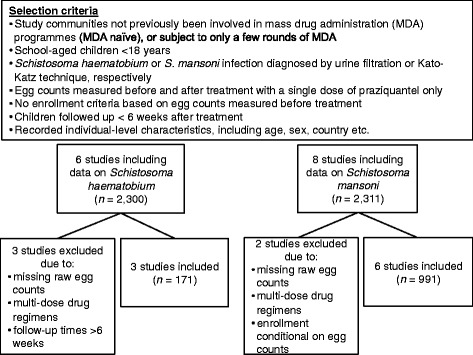
Data selection criteria

**Fig. 2:**
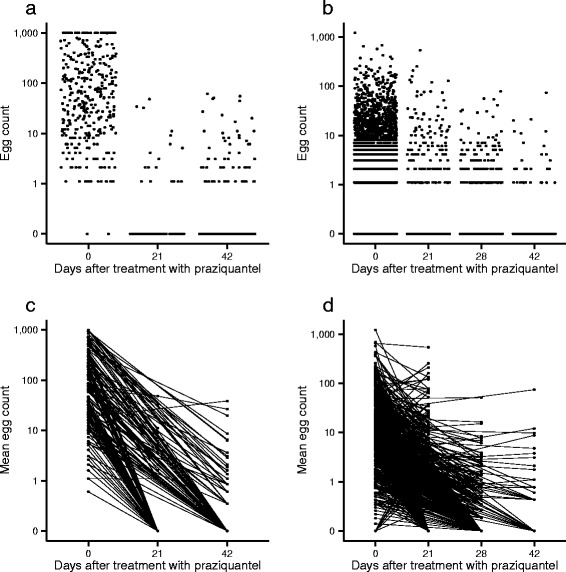
Schistosome egg counts by number of days after treatment with praziquantel. Panel **a** depicts *Schistosoma haematobium* egg counts measured by urine filtration. Panel **b** depicts *S. mansoni* egg counts measured by Kato-Katz technique. Each data point represents a single count (i.e. not an average of multiple counts). Panels **c** and **d** depict the arithmetic mean egg counts per person connected by a line. Treatment with praziquantel occurred following the counts made at day zero

We obtained the datasets presented in Olliaro et al. [14] that comprise individual-level data on schistosome egg counts measured before and after administration of praziquantel, collected from 13 studies (Additional file 1: Supplementary Tables, Table S1). The data also comprise individual covariates including: age; sex; dose of praziquantel, and days of follow-up after treatment, and the population covariate, country. Studies were largely non-controlled, non-blinded public health interventions, with one exception [19], which was placebo-controlled and double blinded. Placebo-controlled randomised controlled trials have been rare for helminthiases as it is now generally considered unethical to withhold effective treatment from infected individuals. Following the criteria outlined in Fig. 1, we selected three studies with data on *S. haematobium* [19–21], and six studies with data on *S. mansoni* [19, 22–26] infections in children. Key features of these datasets are summarised in Table 2 with further details given in Additional file 1: Supplementary Tables, Table S1. Raw egg counts and child-specific mean egg counts before and after treatment with praziquantel are depicted in Fig. 2.

**Table 2:** Summary of data included in the analysis

Country	Participants	Mean age (SD)	QD protocol^a^	PZQ regimen	Follow-up days	Ref.
*Schistosoma haematobium*						
Côte d’Ivoire	6	5 (0)	1 UF × 1 urine sample	1 × 40 mg/kg	21	[20]
Côte d’Ivoire	86	11.1 (1.9)	1 UF × 2 urine samples	1 × 40 mg/kg	21	[21]
Kenya	79	11.3 (3.1)	1 UF × 4 urine samples	1 × 40 mg/kg	42	[19]
*Schistosoma mansoni*						
Côte d’Ivoire	35	3.8 (1.2)	2 KK × 2 stool samples	1 × 40 mg/kg	21	[20]
Uganda	503	4.2 (1.8)	2 KK × 2 stool samples	1 × 40 mg/kg	21	[26]
Côte d’Ivoire	58	11.2 (4.1)	1 KK × 3 stool samples	1 × 40 mg/kg	42	[24]
Côte d’Ivoire	49	8.9 (2.4)	2 KK × 2 stool samples	1 × 40 mg/kg	21	[25]
Côte d’Ivoire	85	10.0 (1.42)	1 KK x 4 stool samples	1 × 40 mg/kg	28	[23]
Côte d’Ivoire	261	9.6 (2.1)	1 KK x 5 stool samples	1 × 60 mg/kg^b^	28	[22]

Abbreviations: *KK* Kato-Katz, *PZQ* praziquantel, *QD* quantitative diagnostic, *SD* standard deviation, *UF* urine filtration

^a^Multiple samples of urine or stool were taken on consecutive days; ^b^ 2 × 30 mg/kg given 3 hours apart

### Model-free approach

We calculated model-free sample ERRs [13] using

1 $$ \mathrm{Sample}\ \mathrm{ERR} = 1\ \hbox{-} \frac{\mathrm{mean}\ \mathrm{egg}\ \mathrm{count}\ \mathrm{after}\ \mathrm{treatment}}{\mathrm{mean}\ \mathrm{egg}\ \mathrm{count}\ \mathrm{before}\ \mathrm{treatment}}, $$

which we compared with model-based estimates. We used a non-parametric percentile block bootstrap method (Table 1) to calculate associated 95 % confidence intervals (CIs). Block bootstrap methods [27] account for correlation among observations (egg counts) from the same individual by randomly sampling (with replacement) *blocks* of data; in this case, all of an individual’s egg counts before and after treatment. Details are given in Additional file 1: *Supplementary Methods S1 Percentile block bootstrap*.

### Modelling approach

We employed marginal models and conditional mixed models [17] to estimate ERRs [16]. These are two distinct classes of statistical regression model suitable for analysing dependent (clustered/correlated) data, which here arises because egg counts are measured repeatedly from the same individual and individuals are sampled repeatedly within the same study. We defined two variants of the latter, one in a classical (frequentist) manner, and the other in a Bayesian framework. Full mathematical details of the models are given in the Additional file 1: *Supplementary Methods* sections *S2 Marginal models*, *S3 Conditional mixed models,* and *S4 Bayesian conditional mixed models*. Here, we give a brief synopsis of the model classes and a description of the salient features for estimating ERRs. Key distinctions are summarised in Table 3.

**Table 3:** Summary of approaches used to estimate egg reduction rates among children infected with schistosomes following treatment with praziquatel

Approach	Method	Required parametric assumptions	Inference
Model-free	Sample statistics	• None	• Population average estimates
Model-based	Marginal models	• Variance to mean relationship • Correlation structure among repeated measures	• Population average estimates • Covariate effects
	Conditional mixed models	• Conditional distribution of data • Distribution of random effects	• Individual estimates • Covariate effects
	Bayesian conditional mixed models	• Conditional distribution of data • Distribution of random effects • Prior distribution of parameters	• Individual estimates • Covariate effects • Fully integrated parameter uncertainty

Marginal models offer population average (marginal) inference, empirically accounting for the dependence of the data using a postulated correlation matrix. In combination with sandwich estimators (Table 1) of coefficient standard errors, marginal models yield robust estimates of uncertainty. Conditional mixed models offer inference at the level of the individual by modelling explicitly the conditional dependence of the data using fixed and random effects. This permits estimation of individual ERRs and the degree of variation among them. By casting conditional mixed models in a Bayesian architecture— defining the necessary parameter prior distributions—one can fully integrate uncertainty into the estimated posteriors and hence derive robust indices of uncertainty, including those associated with the estimated distribution of ERRs among individuals.

The essential ingredient of both model classes (marginal and conditional mixed models) is a log-linear regression structure that describes the change in egg counts after treatment, *x* = 1, compared to before treatment, *x* = 0, in a multiplicative fashion. Hence, the accompanying regression coefficient *β* quantifies the risk ratio (RR) of egg counts after treatment compared to before treatment, and the ERR is given (generically) by 1 – exp(*βx*). Covariates enter the regression structure as *interacting* with *x*. In marginal models this permits ERRs to vary among *strata*. In conditional mixed models, this permits ERRs to vary also among strata, via fixed effects, and additionally among *individuals*, via random effects.

### Inference

We defined marginal and conditional mixed models separately for the *S. haematobium* and *S. mansoni* datasets, including the covariates of ERRs listed in Table 4. In the conditional mixed models, these covariates were treated as exerting both fixed and random effects permitting variation among population strata and among individuals *within* strata. We fitted the models in R [28] using: (a) generalized estimating equation techniques, implemented with the geepack package (marginal models) [29]; (b) restricted maximum likelihood estimation by Laplace approximation, implemented with lme4 (conditional mixed models) [30]; and (c) Markov chain Monte Carlo (MCMC) methods, implemented with MCMCglmm (Bayesian conditional mixed models) [31] (see Table 1 for descriptions of these statistical techniques). We ran three MCMC chains for the Bayesian models, monitoring for convergence and checking that our final conclusions were not dependent on the choice of initial values [32]. In general, 5,000 iterations were discarded as burn-in and an additional 20,000 were sufficient to estimate parameter posterior distributions.

**Table 4:** Covariates included in the regression models used to estimate egg reduction rates among children infected with schistosomes following treatment with praziquatel

Covariate	Levels	
	*Schistosoma haematobium* dataset	*Schistosoma mansoni* dataset
Country	Côte d’Ivoire	Côte d’Ivoire
	Kenya^a^	Uganda
Dose	40 mg/kg	40 mg/kg
		60 mg/kg
Sex	Male	Male
	Female	Female
Age group	Younger SAC^a^	Pre-SAC (<5 years)
	Older SAC	Younger SAC (5–11 years)
		Older SAC (12–17 years)
Follow-up time	21 days^a^	21 days
		28 days
		42 days

Abbreviation: *SAC*, school-age children

^a^Children in Kenya were all followed up for 42 days so follow-up was removed as a covariate

## Results

### Average egg reduction rates

The model-free sample estimates of *S. haematobium* and *S. mansoni* average ERRs following treatment with praziquantel, aggregated across studies, are—with 95 % CIs given in parentheses—99.3 % (98.7 %, 99.7 %) and 83.8 % (77.7 %, 88.9 %), respectively. The corresponding marginal-model estimates (excluding covariates) are 99.6 % (98.1 %, 99.9 %) and 77.9 % (72.7 %, 82.0 %), respectively. The notable difference between the model-free and model-based estimates for *S. mansoni* is because the marginal model accounts for the correlation among the repeated measures. This is compounded by the high variation among the number of observations per individual, which ranged from 4 to 37 [22].

### Stratum average egg reduction rates

**Fig. 3:**
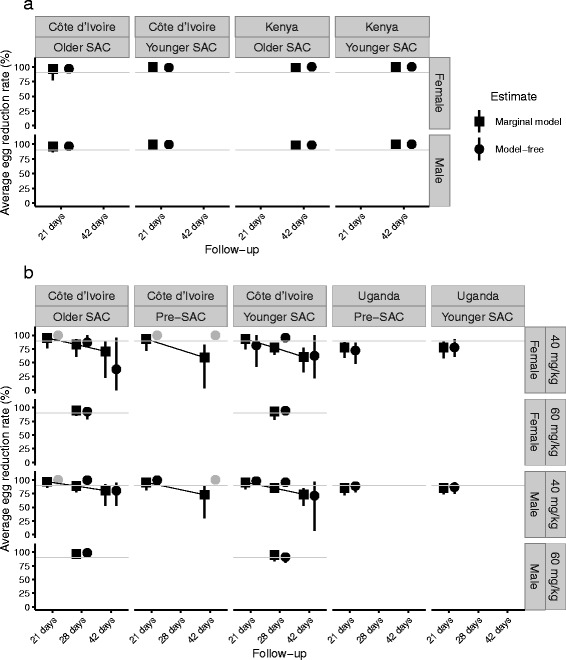
Comparison of egg reduction rates among children infected with schistosomes following treatment with praziquantel estimated by model-free and marginal-model methods. Panels **a** and **b** depict, respectively, estimates from individuals infected with *Schistosoma haematobium* and *S. mansoni.* Subplots within each panel are stratified according to the different covariate combinations defined by the marginal model; some strata are unpopulated and therefore have no data points. Marginal model and model-free estimates are plotted at each follow-up time for ease of visual comparison. Error bars represent 95 % confidence intervals, calculated using bootstrap methods for model-free sample estimates and using robust sandwich estimators of the standard error for marginal-model estimates. Circular data points (depicting model-free estimates) that are coloured grey do not have an associated uncertainty interval since, in the corresponding strata, all egg counts after treatment were zero, and hence are incompatible with the bootstrap approach. The dashed lines in panel **b** highlight the decreasing trend in efficacy for increasing follow-up times as estimated by the marginal model fitted to the *S. mansoni* data (see Fig. 5 for coefficient estimates)

The average ERRs and their accompanying CIs estimated for each stratum (defined by the covariates listed in Table 4) using the model-free and marginal model approaches are depicted in Fig. 3. The two approaches yield similar estimates, albeit the assumptions of the modelling approach stabilise estimates in some poorly populated strata, and generally reduce variation. Moreover, model-free bootstrap CIs cannot be constructed in some strata because no eggs were counted in any samples after treatment. Therefore, in these strata, the estimated ERRs are 100 % with no associated uncertainty (grey circles, Fig. 3b). Some of the model-based average ERRs among children infected with *S. mansoni* fall below the WHO’s 90 % threshold of ‘optimal’ praziquantel efficacy albeit less so at 21 days, which is the WHO-recommended maximum follow-up time (Fig. 3b) [13].

**Fig. 4:**
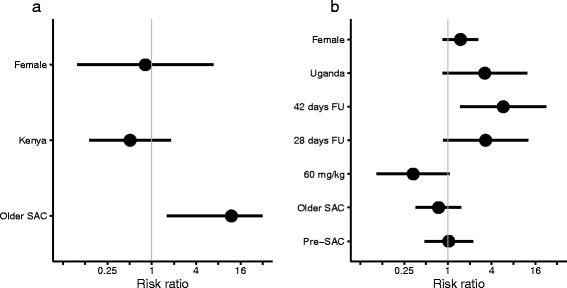
Coefficient estimates of covariates associated with average egg reduction rates among children infected with schistosomes following treatment with praziquantel. Panels **a** and **b** depict coefficients estimated from the marginal models fitted to the data on, respectively, *Schistosoma haematobium* and *S. mansoni* egg counts measured from children before and after treatment with praziquantel. The coefficient point estimates (black circles) indicate the multiplicative change (risk ratio, RR) in egg counts after treatment in a particular covariate group compared to the change after treatment in the reference group. Hence, a RR <1 is associated with an *increased* efficacy and a RR >1 is associated with a *decreased* efficacy (compared with the reference group). Error bars depict 95 % confidence intervals (CIs). A covariate is deemed to exert a statistically significant effect only when its CI does not cross the vertical grey line at RR = 1. For example, older school-aged children (SAC) infected with *S. haematobium* are associated with a statistically significant *decrease* in efficacy (RR >1) compared to younger SAC

**Fig. 5:**
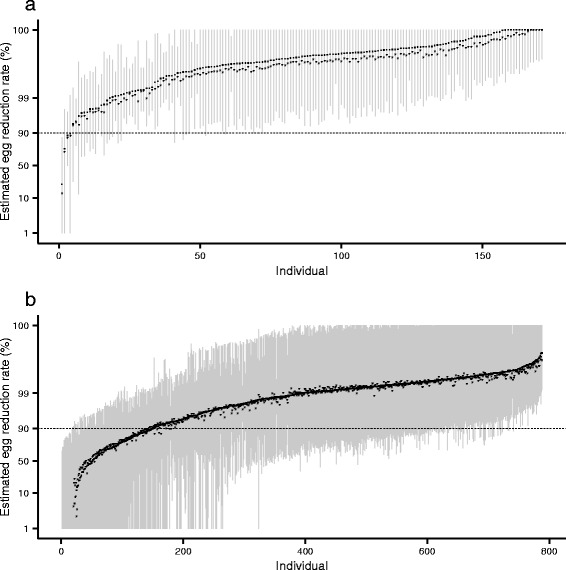
Egg reduction rates among children infected with schistosomes following treatment with praziquantel. Panels **a** and **b** depict, respectively, estimates from children infected with *Schistosoma haematobium* and *S. mansoni.* Egg reduction rates are calculated from the empirical best linear unbiased predictors (see Table 1 for definition) estimated from the classical (frequentist) conditional mixed models. Negative estimates of ERRs (**a**: *n* = 1, 0.59 %; **b**: *n* = 24, 2.4 %), which correspond to an increase in egg counts after treatment compared to before treatment, are not shown

Underlying the marginal-model estimates shown in Fig. 3 are the estimated covariate coefficients. These are presented in the form of RRs in Fig. 4, alongside accompanying 95 % CIs. We also present the ERRs corresponding to these RRs in Table 5. The estimates in Fig. 4a indicate that average *S. haematobium* egg counts from older SAC (Fig. 5a) after treatment *relative to before treatment* are approximately 12 times greater than those from younger SAC (*P*-value = 0.016). However, this seemingly pronounced difference corresponds to an average ERR of 95.9 % (85.8 %, 98.8 %) compared to 99.7 % (99.4 %, 100 %), respectively (with other covariates set to their baseline values, i.e. males from Côte d’Ivoire, Table 5).

**Table 5:** The effect of covariates on average egg reduction rates among children infected with schistosomes following treatment with praziquantel

Covariates	Levels	ERR (95 % CI)
*Schistosoma haematobium* dataset		
(Baseline)	Côte d’Ivoire; male; younger SAC; 40 mg/kg; 21 days follow-up	99.7 % (97.4 %, 100 %)
Country	Kenya^a^	99.8 % (98.8 %, 100 %)
Sex	Female	99.7 % (95.7 %, 100 %)
Age group	Older SAC	95.9 % (85.8 %, 98.8 %)
*Schistosoma mansoni* dataset		
(Baseline)	Côte d’Ivoire; male; younger SAC; 40 mg/kg; 21 days follow-up	95.4 % (83.1 %, 98.8 %)
Country	Uganda	85.3 % (73.5 %, 91.8 %)
Dose	60 mg/kg	98.5 % (90.8 %, 99.8 %)
Sex	Female	93.2 % (74.1 %, 98.2 %)
Age group	Pre-SAC	95.3 % (80.9 %, 98.8 %)
	Older SAC	96.6 % (85.8 %, 99.2 %)
Follow-up time	28 days	84.9 % (76.7 %, 90.1 %)
	42 days	73.6 % (53.2 %, 85.1 %)

Abbreviations: *CI* confidence intervals, *SAC* school-age children

^a^Children in Kenya were followed up for 42 days

The estimates in Fig. 4b highlight the increasing trend in the RRs from 21 days to 28 days to 42 days, corresponding to a *decreasing trend* in the average ERR (as also evident by the trend lines in Fig. 3b). Egg counts made at 42 days after treatment *relative to before treatment* were 5.77 times greater than those made at 21 days (*P-*value = 0.012), corresponding to ERRs of 73.6 and 95.4 % respectively (Table 5). Also noteworthy is that average egg counts following a 60 mg/kg oral dose of praziquantel are 66.8 % lower compared with a 40 mg/kg dose (*P-*value = 0.064), corresponding to a (not statistically significant) *increase* in the ERRs from 95.4 to 98.5 %, (with other covariates held at their baseline values, i.e. male younger SAC in Côte d’Ivoire, Table 5).

### Individual egg reduction rates

In Fig. 5 we show individual ERRs across all studies, adjusted for covariate fixed effects and estimated by the classical (frequentist) and Bayesian conditional mixed models for *S. haematobium* (Fig. 5a) and *S. mansoni* (Fig. 5b). The point estimated ERRs (so-called empirical best linear unbiased predictors, EBLUPs, Table 1) from the classical conditional mixed models (denoted by stars in Fig. 5) indicate that the percentage of individuals with an ERR of greater than 90 % is 97.7 and 80.7 % for *S. haematobium* and *S. mansoni* respectively. The corresponding percentages calculated using the Bayesian posterior medians (denoted by dots in Fig. 5) are 96.5 and 80.8 %. However, when the uncertainty in the estimated ERRs is taken into account—by calculating the fraction of individuals with an ERR > 90 % for each draw from the estimated parameter posterior—the median percentages and associated Bayesian credible intervals (BCIs, given in parentheses) are 97.1 % (94.2 %, 98.8 %) and 75.9 % (67.0 %, 81.0 %) for *S. haematobium* and *S. mansoni* respectively.

**Fig. 6:**
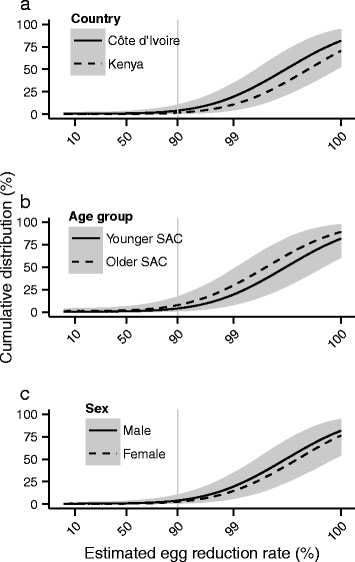
Cumulative distributions of egg reduction rates among children infected with *Schistosoma haematobium* following treatment with praziquantel. Cumulative distributions (black lines) are constructed from the posterior distributions of the fixed and random effects components of egg reduction rates estimated from the Bayesian conditional mixed models. Distributions are depicted by country, age group and sex in panels **a**, **b** and **c** respectively. In all panels, covariates not indicated in the legend are set to their baseline levels, i.e. male younger school-aged children from Côte d’Ivoire followed up after 21 days, see Table 4. Grey shaded areas depict 95 % Bayesian credible intervals

**Fig. 7:**
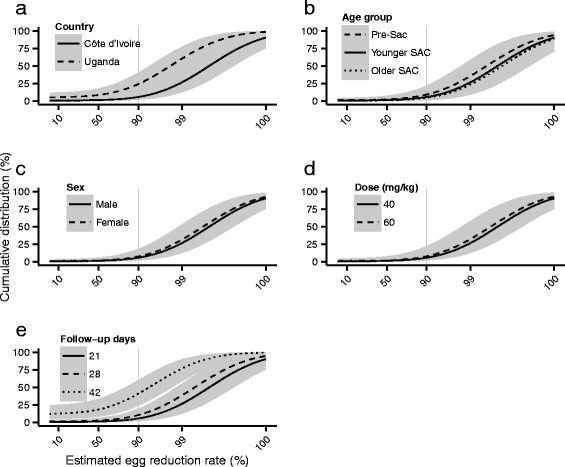
Cumulative distributions of egg reduction rates among children infected with *Schistosoma mansoni* following treatment with praziquantel. Cumulative distributions (black lines) are constructed from the posterior distributions of the fixed and random effects components of egg reduction rates estimated from the Bayesian conditional mixed models. Distributions are depicted by country, age group, sex, dose and follow-up days in panels **a**, **b**, **c**, **d** and **e** respectively. In all panels, covariates not indicated in the legend are set to their baseline levels, i.e. male younger school-aged children given 40 mg/kg praziquantel from Côte d’Ivoire followed up after 28 days, see Table 4. Grey shaded areas depict 95 % Bayesian credible intervals

The cumulative distributions (percentiles) of individual ERRs *within* strata, estimated from the Bayesian conditional mixed models, are depicted in Figs. 6 and 7 for *S. haematobium* and *S. mansoni* respectively. The corresponding fractions of individual responses greater than 90 % are given in Table 6. The distributions of ERRs, like the point estimates shown in Fig. 5, show that praziquantel is highly efficacious in the majority of children (ERRs > 90 %) but that a substantial minority have ERRs below the 90 % threshold. In particular, the distributions estimated from children infected with *S. mansoni* in Uganda, or from those followed-up after 42 days, have longer left tails and greater uncertainty than those estimated from children in Côte d’Ivoire or followed-up after a shorter duration. In Uganda, the median and 95 % BCI associated with the proportion of children with an ERR greater than 90 % is 75.9 % (59.7 %, 86.4 %) compared to 94.4 % (85.3 %, 98.3 %) in Côte d’Ivoire. The corresponding median and 95 % BCI associated with a 42-day follow up is 59.1 % (39.7 %, 76.4 %) compared to 94.4 % (85.3 %, 98.3 %) with a 21-day follow-up.

**Table 6:** The effect of covariates on the percentage of egg reduction rates greater than 90 % among children infected with schistosomes following treatment with praziquantel

Covariates	Levels	Percentage ERR > 90 % (95 % BCI)
*Schistosoma haematobium* dataset		
(Baseline)	Côte d’Ivoire; male; younger SAC; 40 mg/kg; 21 days follow-up	95.9 % (89.3 %, 99.1 %)
Country	Kenya^a^	98.3 % (94.3 %, 99.7 %)
Sex	Female	97.3 % (92.2 %, 99.4 %)
Age group	Older SAC	92.3 % (82.6 %, 97.6 %)
*Schistosoma mansoni* dataset		
(Baseline)	Côte d’Ivoire; male; younger SAC; 40 mg/kg; 21 days follow-up	94.4 % (85.3 %, 98.3 %)
Country	Uganda	75.9 % (59.7 %, 86.4 %)
Dose	60 mg/kg	92.3 % (77.8 %, 98.1 %)
Sex	Female	92.6 % (81.8 %, 97.6 %)
Age group	Pre-SAC	90.9 % (78.1 %, 97.2 %)
	Older SAC	95.5 % (85.8 %, 99.0 %)
Follow-up time	28 days	89.5 % (80.4 %, 95.0 %)
	42 days	59.1 % (39.7 %, 76.4 %)

^a^Children in Kenya were followed up for 42 days

Abbreviations: *BCI* Bayesian credible interval, *SAC* school-age children

## Discussion

Modelling methods are currently underused for assessing anthelminthic drug efficacy. By analysing longitudinal data on the intensity of schistosome infections before and after treatment with praziquantel, we show that marginal and conditional mixed models can be used to obtain robust estimates of both population- and individual-level efficacies, while concurrently evaluating the effects of covariates. While a small number of anthelminthic drug studies have employed various somewhat unconventional approaches [33–35], statistical modelling techniques have not translated into more general use in either the veterinary or human fields (but see [36]). Beyond the realm of estimating anthelminthic drug efficacy, longitudinal and hierarchical modelling techniques are often used incorrectly across a variety of disciplines in ecology and evolutionary biology [37], indicating a general lack of applied understanding about these powerful analytical tools.

### The efficacy of praziquantel within populations and among individuals

Model-free sample-based methods for estimating anthelminthic drug efficacy are hindered by their inherent inefficiency in handling covariates (although we note that the WHO protocol for measuring ERRs recommends evaluating ERRs at a standardized 21-day follow-up in SAC using a single test on a single sample before and after treatment) [16]. Moreover, they can produce biased estimates when individuals are assessed with different numbers of repeated measures (e.g. different numbers of Kato-Katz counts per stool sample, or different numbers of stool samples, either before or after treatment). This is because all observations are weighted equally and, therefore, individuals contributing more observations disproportionately influence the estimated statistic [38], i.e. the sample ERR. This probably explains some of the differences between the model-free and marginal-model estimates of ERR presented here. Confidence intervals associated with average ERRs estimated using the marginal models capture the effect of correlated repeated measures using robust sandwich estimators of the standard errors (Table 1). Although the block bootstrap method used to construct CIs for the model-free approach accounts for correlation in a more rudimentary fashion, it is inappropriate in strata when no egg counts are observed after treatment, generating a statistically invalid CI with a width of zero and a 100 % point-estimated ERR (grey circles in Fig. 3b).

The estimated ERRs of praziquantel against *S. haematobium* from both the model-free and marginal modelling approaches were noticeably higher than those for *S. mansoni* (Fig. 4). It is possible that this difference is driven by a mechanistic superiority of praziquantel against *S. haematobium*, for which there is some supportive in vitro evidence [39]. It is also possible that the difference is driven by a differential distribution of praziquantel to the parts of the perivesical venous plexus and the mesenteric/rectal veins where adult *S. haematobium* and *S. mansoni* flukes reside, respectively [40, 41]. Another possibility is that the discrepancy in ERRs reflects the presence of praziquantel-tolerant *S. mansoni*, which albeit rare, has been previously observed in the field [12]. By contrast, tolerant *S. haematobium* specimens have not been [8], notwithstanding the reports of individuals requiring multiple doses of praziquantel to clear infection [42, 43].

Perhaps most pertinent to the inter-species difference is that the modelling approaches illustrated here do not account for the sensitivity of the diagnostic method (Kato-Katz thick smear for *S. mansoni* and urine filtration for *S. haematobium* diagnosis) and, in particular, the manner in which sensitivity declines with decreasing infection intensity, before and after praziquantel administration [44, 45]. This phenomenon can cause overestimation of drug efficacy [23, 46] with differential bias between species if the severity of this effect is different between the urine filtration technique and the Kato-Katz method. Moreover, it is assumed that the specificity of the diagnostic methods are 100 %, with no incorrect identification of schistosome eggs in truly negative samples. In particular, as the intensity of infection declines after treatment, the number of true negative samples increases, increasing the probability of false positives, even with very good specificity. The next generation of modelling frameworks must account for the sensitivity and specificity of the diagnostic method and capture how it varies with the underlying intensity of infection.

While marginal models are powerful tools for assessing stratum-level average efficacy (average ERRs), conditional mixed models offer additional insight at the level of the individual. The individual estimates of ERRs reveal numerous so-called sub-optimally responding individuals, with an efficacy lower than the WHO’s empirical 90 % threshold for group average. A similar result is presented in the recent sample-based analysis of the full 13-study database (see Additional file 1: Supplementary Tables, Table S1) [14]. A substantive 3.7 % of children infected with *S. mansoni* had ERRs of less than 50 % (Fig. 6) and more than twice as many children infected with *S. mansoni* had an ERR below 90 % compared with those infected with *S. haematobium* (10.4 % *versus* 4.0 %). Suboptimal or atypical responses to praziquantel have been previously described in terms of the number of doses necessary to clear the parasite, with any result above one dose considered suboptimal [42, 43, 47–49]. Compared to this measure of sub-optimality, conditional mixed models offer a more nuanced insight into the distribution of drug responses among individuals.

### When does the efficacy of praziquantel appear greatest?

The observed efficacy of an anthelminthic crucially depends on when after treatment measurements of infection intensity (or presence/absence of transmission stages) are made. This is because efficacy is a snapshot of the competing dynamics of parasite clearance, followed by reinfection [50] or repopulation initiated by surviving parasites, perhaps subjected to temporarily reduced or inhibited fertility or, in the case of praziquantel, maturation of juvenile parasites [45].

Our results indicate that, among children infected with *S. mansoni*, average ERRs decreased from follow-up times of 21 days to 28 days, and from 28 days to 42 days. This mirrors the findings of a meta-analysis of 11 studies of the efficacy of praziquantel against *S. mansoni* in Africa where a similar relationship between longer follow-ups and lower CRs was interpreted as being probably due to reinfection [48]. Repopulation by surviving juvenile parasites is another likely cause. Schistosomes exhibit a biphasic susceptibility to praziquantel throughout their lifecycle; early-stage migrating larvae are susceptible to praziquantel, but after about one month of growth, susceptibility drops precipitously, and is only regained after another two months [51]. Therefore, by 42 days after treatment, many of the juvenile schistosomes that survived treatment may have matured or *repopulated* the organs of preferred location as fertile egg-producing adult schistosomes. Moreover, recent studies using circulating cathodic antigen (CCA) tests indicate that 50–100 % of praziquantel-treated children retain their antigenemia/antigenuria when retested 1–7 weeks after treatment [52, 53]. Hence, the effective praziquantel efficacy is probably much lower than previously thought and surviving parasites are also likely contributors to the recovery in egg counts after treatment. We did not have access to data collected at shorter follow-up times, to perhaps model when ERRs are at a maximum, although analyses presented elsewhere [23] have suggested that this occurs 2–3 weeks after treatment, which is the timeframe currently recommended by WHO [13].

### Why does age affect the efficacy of praziquantel?

The decreasing (weak and not statistically significant) trend in estimated ERRs with increasing age (pre-SAC to younger SAC to older SAC, see Figs. 5b and 7c) of children infected with *S. mansoni* is in accordance with several existing hypotheses. Older children are likely to have stronger acquired immunity to schistosomes than younger children [54], as flukes killed or damaged from exposure to praziquantel release previously ‘unseen’ antigens [55–58] eliciting protective immune responses thought to enhance (but not always [59]) the efficacy of subsequent treatments [60, 61]. Consequently, one might expect older children to be more amenable to treatment and exhibit higher ERRs than their younger counterparts. However, and crucially, the data analysed here were collected from communities unexposed or minimally exposed to praziquantel MDA (see selection criteria in Fig. 1). Hence, older children in these communities might be more difficult to treat and respond less well to praziquantel having been left to grow older with untreated schistosome infections. This might explain the estimates from the marginal model indicating that the average ERR from older SAC infected with *S. haematobium* is markedly lower than that from younger SAC. Alternatively, this result may reflect a general limitation of the analysis. For example, most of the studies under consideration encompassed multiple villages or study areas within a single country, but village-level identifiers were not available, and small-scale variation in praziquantel responses among villages can be quite large [62].

### How does infection intensity affect the efficacy of praziquantel?

Numerous studies have demonstrated a negative association between the infection intensity before treatment and estimated CRs following treatment with praziquantel; the higher the intensity, the lower the CR [20, 48, 63]. This is because, if adult schistosomes die with a fixed probability when exposed to praziquantel, cure will be less likely in heavily infected individuals than in lightly infected individuals. Indeed, the WHO no longer recommends using CRs for the monitoring and evaluation (M&E) of anthelmintic efficacy [13] because it is impossible to observe incremental reductions in parasite burden using a binary measure of cure and, therefore, CRs fail to capture the impact of multiple doses of anthelminthic drugs over the course of PCT programmes [64]. In this work, we constructed the conditional mixed models to estimate the association between an individual’s egg count before treatment (the random intercept term) and their ERR (governed by the random ‘gradient’ term, see Additional file 1: *Supplementary Methods,S3 Conditional mixed models*), but we found no statistically significant relationship. Intuitively, again invoking the assumption of a constant probability of death by praziquantel, the percentage reduction in intensity will be constant, explaining why there is no association between egg counts before treatment and the estimated ERR.

### Modelling for M&E of MDA interventions

Modelling has an important role in the M&E of anthelmintic drug efficacy in the context of human helminth PC programmes. Marginal models offer a robust method of estimating (sub-) population-level drug efficacy, which would allow disease control managers to identify whether or not target ERRs are being met. Such targets could be defined using data from populations predominantly naïve to MDA, in different demographic groups, which may respond in a systematically differential manner to drug treatment. Individual-level ERRs estimated using conditional mixed models offer an additional depth of insight, permitting characterisation of the distribution of drug responses among individuals. This is important for the rapid identification of changing responses to anthelminthic drugs that may be indicative of declining drug efficacy, potentially caused by emerging drug-resistant parasites [6, 10–12, 49]. By comparing the observed distribution of drug responses to a reference distribution of expected responses estimated before MDA—ideally from the same community, but otherwise using data from demographically and geographically matched or partially matched populations—it would be possible to identify whether or not individuals are responding aberrantly to the drug. That is, one could quantify how atypical an observed response is compared to the usual or expected distribution of responses. Individuals responding suspiciously could be investigated, perhaps using in vitro drug sensitivity tests on the infecting parasites. Moreover, over multiple rounds of MDA, one could identify shifts in the distribution of responses from the original reference distribution; shifts towards decreased efficacy triggering further programmatic and parasitological investigation.

## Conclusions

Marginal and conditional mixed models are robust approaches for calculating population- and individual-level estimates of anthelminthic drug efficacy. We illustrate these techniques by analysing data collated from nine previous studies on schistome egg counts from children before and after administration of praziquantel. We show that model-based analyses: (a) offer more stable and robust estimates of average ERRs compared to traditional sample-based methods, especially when sample sizes are small; (b) can be used to evaluate how and to what degree drug responses vary among population strata, in terms of an average response, and among individuals within a stratum, in terms of the distribution of individual responses. We show that Bayesian methods are particularly useful in quantifying uncertainties, and permit creation of prototype ‘reference’ distributions describing the range of drug responses expected in communities predominantly naïve to MDA. These distributions have potentially important applications to the M&E of anthelmintic efficacy in helminthiasis PCT programmes, particularly for identifying individual atypical responses and distributional shifts, potentially indicative of emerging drug resistance. Therfore, the approaches illustrated in this paper have an important role in supporting the control and elimination of human helminthiases.

## Additional file

Additional file 1:
**Supplementary Methods, Figures and Tables.** (DOCX 108 kb)

## Abbreviations

BCI: Bayesian credible interval
CCA: circulating cathodic antigen
CI: confidence interval
CR: cure rate
ERR: egg reduction rate
GEE: generalized estimating equation
GLM: generalized linear model
GLMM: generalized linear mixed model
IRR: intensity reduction rate
M&E: monitoring & evaluation
MCMC: Markov chain Monte Carlo
MDA: mass drug administration
NTD: neglected tropical disease
PCT: preventative chemotherapy
SAC: school-aged children
WHO: World Health Organization
