# Supplementary material for: New approaches to measuring anthelminthic drug efficacy: parasitological responses of childhood schistosome infections to treatment with praziquantel
Source: Parasit Vectors. 2016 Jan 27;9:41. doi: 10.1186/s13071-016-1312-0 (PMC4728951; doi:10.1186/s13071-016-1312-0)
Supplement: Additional file 1: — Supplementary Methods, Figures and Tables. (DOCX 108 kb) [file 13071_2016_1312_MOESM1_ESM.docx]

**Supplementary Material**

**New approaches to measuring anthelminthic drug efficacy: parasitological responses of childhood schistosome infections to treatment with praziquantel**

Martin Walker^1,2,*§^, Tarub S. Mabud^1,‡,*^, Piero L. Olliaro^3,4^, Jean T. Coulibaly^5,6,7,8^, Charles H. King^9,10^, Giovanna Raso^5,6^, Alexandra U. Scherrer^11^, J. Russell Stothard^12^, José Carlos Sousa-Figueiredo^13,14^, Katarina Stete^15^, Jürg Utzinger^5,6^, Maria-Gloria Basáñez^1,2^

^1^ Department of Infectious Disease Epidemiology, School of Public Health, Faculty of Medicine (St Mary’s Campus), Imperial College London, London W2 1PG, United Kingdom

^2^London Centre for Neglected Tropical Disease Research, Department of Infectious Disease Epidemiology, Imperial College London, London W2 1PG, United Kingdom

^3^UNICEF/UNDP/World Bank/WHO Special Programme on Research and Training in Tropical Diseases (TDR), World Health Organization, Av. Appia 20, CH-1211 Geneva 27, Switzerland

^4^ Centre for Tropical Medicine and Global Health Nuffield Department of Medicine Research Building University of Oxford Old Road Campus, Roosevelt Drive, Oxford OX3 7FZ, United Kingdom

^5^Department of Epidemiology and Public Health, Swiss Tropical and Public Health Institute, P.O. Box, CH-4002 Basel, Switzerland

^6^University of Basel, P.O. Box, CH-4003 Basel, Switzerland

^7^Unité de Formation et de Recherche Biosciences, Université Félix Houphouët-Boigny, 22 BP 770, Abidjan 22, Côte d’Ivoire

^8^ Centre Suisse de Recherches Scientifiques en Côte d’Ivoire, 01 BP 1303, Abidjan 01, Côte d’Ivoire

^9^Center for Global Health and Diseases, Case Western Reserve University, 2109 Adelbert Road, Cleveland, OH 44106, United States of America

^10^ Schistosomiasis Consortium for Operational Research and Evaluation, University of Georgia, 500 D W Brooks Drive, Athens, GA 30602, United States of America

^11^Division of Infectious Diseases and Hospital Epidemiology, University Hospital Zurich, University of Zurich, Rämistrasse 100, CH-8091 Zurich, Switzerland

^12^Department of Parasitology, London School of Tropical Medicine, Liverpool L3 5QA, United Kingdom

^13^Centro de Investigação em Saúde de Angola (Health Research Center in Angola), Rua direita do Caxito, Hospital Provincial, Bengo, Angola

^14^ Department of Life Sciences, Natural History Museum, Wolfson Wellcome Biomedical Laboratories, Cromwell Road, London, SW7 5BD, United Kingdom

^15^Center for Infectious Diseases and Travel Medicine, Department of Medicine, University Hospital Freiburg, Hugstetter Strasse 55, D-79106 Freiburg, Germany

^‡^ Present address: Stanford School of Medicine, 291 Campus Drive, Stanford, CA 94305, United States of America

*Contributed equally to this work

^§^Corresponding author

Email addresses:

MW: [m.walker06@imperial.ac.uk](mailto:m.walker06@imperial.ac.uk)

TSM: [tarub.mabud@gmail.com](mailto:tarub.mabud@gmail.com)

PLO: [olliarop@who.int](mailto:olliarop@who.int)

JTC: [couljeanvae@yahoo.fr](mailto:couljeanvae@yahoo.fr)

CHK: chk@case.edu [chk@case.edu](mailto:chk@case.edu)

GR: [giovanna.raso@unibas.ch](mailto:giovanna.raso@unibas.ch)

AUS: [alexandra.scherrer@usz.ch](mailto:alexandra.scherrer@usz.ch)

JRS: [russell.stothard@lstmed.ac.uk](mailto:russell.stothard@lstmed.ac.uk)

JCSF: [josecarlos.figueiredo@gmail.com](mailto:josecarlos.figueiredo@gmail.com)

KS: [kata.stete@gmail.com](mailto:kata.stete@gmail.com)

JU: [juerg.utzinger@unibas.ch](mailto:juerg.utzinger@unibas.ch)

MGB: [m.basanez@imperial.ac.uk](mailto:m.basanez@imperial.ac.uk)

**Supplementary Methods**

## S1 Percentile block bootstrap

The percentile block bootstrap approach [1] to calculate 95% confidence intervals (CIs) associated with sample egg reduction rates (ERRs) was implemented as follows:

1. Sample with replacement *n* vectors (collections) of egg counts measured before *and* after treatment from individual children.
2. Calculate the sample ERR from the new sample of egg counts using Equation (1) in the main text.
3. Repeat steps (i) and (i) 10,000 times to yield an empirical sampling distribution of the (sample) ERR.
4. Calculate CIs from the 2.5% and 97.5% percentiles of the (sample) ERR sampling distribution.

## S2 Marginal models

Marginal models are closely related to generalized linear models (GLMs), but by modelling the correlation among dependent data, they are suitable for analysing (longitudinal) repeated measures [2]. Like GLMs, marginal models can be formulated to yield stratum-specific estimates; here, average ERRs within groups of children sharing sets of covariates. Unlike GLMs, these estimates and their accompanying uncertainties are appropriately adjusted for non-independent (dependent) data. In addition to the (log-linear) regression structure, marginal models comprise two key components: the correlation structure and the variance of the data, the latter typically specified as a function of the mean (see Table 1 and Table 3 in the main text).

We defined parameters to capture correlation among egg counts from the same child. We defined: (a) cross-sectional correlation, among egg counts collected *either* before *or* after treatment, and (b) longitudinal correlation, among egg counts collected before *and* after treatment, and specific to each follow-up time. We found this structure sufficient to capture the substantive effects of correlation among repeated measures from the same child; we did not attempt to capture explicitly correlation among egg counts collected from different children within the same study nor did we try to capture possibly distinct correlations among counts measured (from the same child) on the same day compared to on different days (both these processes are explicitly modelled in the conditional mixed model framework, see *S3 Conditional mixed models*).

We assumed that the marginal variance (the variance among observations from a single stratum, i.e. sharing a common set of covariates) of the egg counts was overdispersed relative to the variance of the Poisson distribution. This was modelled using a scale parameter that permitted the variance to vary linearly with the mean, rather than being equal to the mean, which is the case for Poisson-distributed counts.

The marginal model is defined as

**μ***_i_* = exp(**βX**^T^*_i_*),

**ν***_i_* = *ϕ***μ***_i_*,

Corr(**y***_i_*) = **R***_i_* (S1)

where **μ***_i_* and **v***_i_* are vectors of (modelled) means and variances for the collection of observed egg counts from individual *i*, **y***_i_*. Each element of **μ***_i_* and **v***_i_*, denoted *μ_ij_* and *v_ij_* respectively, correspond to a particular egg count *j*, with *v_ij_* modelled as a linear function of *μ_ij_* with coefficient *ϕ* correspond to the degree of extra-Poisson variation or overdispersion. The matrix **X**^T^*_i_* is the transpose (indicated by the superscript T) of the design matrix, **X***_i_*, for individual *i*. Each row of **X***_i_* comprises a vector of covariates indicating whether a particular observation was made before or after treatment *and its interactions* with the following factors: (i) study country; (ii) patient age, categorised nominally as preschool-aged children (pre-SAC, <5 years), younger SAC (5-11 years) and older SAC (12-17 years); (iii) sex; (iv) follow-up duration (defined as a categorical variable); and (v) praziquantel dose (categorical). The regression coefficients **β** relate the covariate information contained within **X***_i_* to the vectors of means and variances **μ***_i_* and **v***_i_*, respectively. Matrix **R***_i_* is a correlation matrix comprising off-diagonal correlation coefficients defining the correlation structure among egg counts measured from the same individual *i.* For example, for an individual contributing two egg counts before treatment, *y*_1_*_i_* and *y*_2_*_i_*, and two egg counts after treatment, *y*_3_*_i_* and *y*_4_*_i_*, **R***_i_* takes the form

(S2)

where *ρ*_1_ and *ρ_i_* refer to, respectively, cross-sectional correlation (among egg counts collected *either* before *or* after treatment) and longitudinal correlation (among egg counts collected before *and* after treatment) from the same individual. Correlation *ρ_i_* is subscripted to reflect that egg counts were measured from individuals at variable follow-up times after treatment. For example, to model the *S. haematobium* dataset, we defined three correlation parameters: *ρ*_1_, defining cross-sectional correlation among repeated measures before treatment, follow-up day 21 and follow-up day 42; *ρ*_2_, defining the longitudinal correlation before treatment and follow-up day 21; and *ρ*_3_, defining the longitudinal correlation before treatment and follow-up day 42.

## S3 Conditional mixed models

Conditional models account for correlation among longitudinal and hierarchically structured data by modelling the observations *conditional* on unit-specific random effects terms [2]. Here we consider three units of clustering: (i) observations (egg counts) from children within the same *study*; (ii) observations made at different times from the same *child*, and (iii) observations made on the same *day* (repeated egg counts were made from stool or urine samples collected on consecutive days before and after treatment, see Table 2 in the main text or Table S1)*.* Conditional mixed models comprise fixed and random effects, and are suitable for modelling non-normally distributed data. The simplest model incorporates a random effects adjustment of the population or ‘global’ intercept coefficient, the so-called random intercepts model. More complex models can be constructed whereby the coefficients of any covariates are permitted to vary among units. For example, by allowing **β** (the coefficients of **x***_i_*) to vary among children using a set of adjustments **b***_i_*, we obtain child-specific estimates of praziquantel efficacy which vary among children within the same stratum (i.e. sharing a common set of covariates),

Model ERR*_i_* = 1 – exp[**βx***_i_* + **b***_i_*]. (S3)

In addition to the (log-linear) regression structure, mixed models require specification of: (a) the assumed distribution of the data; (b) which coefficients are to be considered as exerting random effects; and (c) how the random effects are distributed among units (children and studies).

We define the conditional mixed model as

*y_ij_*~ Poisson(*μ_ij_*),

**μ***_i_* = exp(**βX**^T^*_i_* + **b***_i_***Z**^T^*_i_*+ **e***_i_*),

**b***_i_* ~ MVN(**0**, **Σ**),

*e_ij_* ~ N(0,*σ*). (S4)

Here, *y_ij_* denotes egg count *j* measured from individual *i* which is assumed to be a realization from a Poisson a distribution with mean *μ_ij_*, *conditional on* the fixed and random effects covariate values contained within the matrices, **X***_i_* and **Z***_i_*, respectively, and their accompanying coefficients **β** and **b***_i_*. The fixed effects covariates included in **X***_i_* are identical to those specified in the marginal models (see *S2 Marginal models*). The random effects covariates within **Z***_i_* include intercept terms for the day of measurement, the individual child and the particular study, as well as an individual ‘gradient’ term for the effect of treatment. The random effects coefficients **b***_i_* are assumed to follow a multivariate normal distribution (MVN) with means equal to zero and a variance-covariance matrix **Σ** with dimensions equal to the number of covariates specified as exerting random effects (i.e. the length of each row of **Z***_i_*). Finally, the model includes an additional vector of random effects, **e***_i_*, which are specific to each observation and permit extra-Poisson variation (overdispersion) for each *y_ij_*| *μ_ij_* [3-5]. That is, the distribution of egg counts from the same individual measured on the same day can be overdispersed (Poisson-lognormal) with variance greater than the mean, as opposed to the more restrictive Poisson assumption where the variance is equal to the mean. Each *e_ij_* in Equation (S3) is assumed normally distributed (on the logarithmic scale) with mean 0 and variance *σ*^2^ quantifying the degree of overdispersion.

Matrix **Σ** has two features which are particularly important in the context of this analysis. First, a subset of the diagonal elements of **Σ** define variation among individual responses to treatment (i.e. individual ‘gradients’ associated with treatment) and—combined with the corresponding fixed-effect elements of **β** and the underlying assumption that the random effects follow a multivariate normal distribution on the log-scale—permit construction of estimated distributions of ERRs within population strata (defined by the categories of covariates within **X***_i_*). Second, the off-diagonal elements of **Σ** define the covariance (correlation) between children’s responses to treatment with praziquantel and their intensity of infection (measured by egg counts) before treatment. This provides information on whether or not the efficacy of praziquantel is density dependent. For example, a positive correlation implies that higher than average egg counts before treatment are associated with higher (less negative) effects or ‘gradients’ of treatment and lower ERRs, indicative of reduced praziquantel efficacy. Conversely, a negative correlation implies that lower than average egg counts before treatment are associated with lower (more negative) ‘gradients’ of treatment and higher ERRs, indicative of elevated praziquantel efficacy.

## S4 Bayesian conditional mixed models

The Bayesian GLMM used for estimating the efficacy of PZQ on an individual basis *and* permitting robust inference on the variance-covariance matrix **Σ** (see main text Models and Methods, *Bayesian generalized linear mixed model*) has an identical structure to the frequentist GLMM defined in Equation (S3). The only additional requirement to cast the model in a Bayesian character is the specification of parameters prior distributions (priors). We specified the following uninformative (vague, diffuse) prior distributions:

1. Normal distributions with nominally large variance for the fixed effect covariate coefficients, **β**.
2. An inverse-Wishart distribution, W^-1^(**V**, *η*), for the random effects variance-covariance matrix **Σ**, where **V** is a diagonal matrix with diagonal entries equal to 1 (and off-diagonals equal to 0 implying *a priori* uncorrelated random effects) and *η =*0.02. This parameterization of the inverse-Wishart distribution—which is conjugate to the variance-covariance matrix of a multivariate-normal distribution (i.e. the distribution of **b***_i_*) [6]—is suitably uninformative; *η* represents one’s confidence that **V** is the actual variance-covariance, so small values of *η* imply large uncertainty [7].
3. An inverse-gamma distribution, G^-1^(*α*, *γ*), for each observation-specific random effect *e_ij_* with shape, *α* = *η*/2 and scale *γ* = 1 / 2 corresponding to the univariate special case of the inverse-Wishart distribution W^-1^(**V**, *η*).

## S5 Interpreting coefficients

The fixed effects coefficient estimates (or their posteriors from the Bayesian analysis) associated with ERRs (i.e. the coefficients of the relevant interaction terms) have a different interpretation when estimated from the conditional mixed models compared to the marginal models. In the population-based marginal models, the relevant coefficients quantify the association between a covariate and the *average ERR* among children. By contrast, in the individual-based conditional mixed models, the relevant fixed effects coefficients quantify the association between a covariate and the ERR of a *typical or average* child. These estimates (which are presented in Figure S1 and are reassuringly similar between the classical and Bayesian model variants) are less intrinsically interesting than the coefficient estimates from the marginal model, and are rather better interpreted by the manner in which they influence the *distribution* of ERRs, as presented in Figure 6 and Figure 7 in the main text. For example, the risk ratios associated with a typical child infected with *S. mansoni* are substantially higher (and strongly statistically significant) in Uganda compared to Côte d’Ivoire, and after 42 days of follow-up compared with 28 days of follow-up (Figure S1). Although these differences translate into only modest differences in the ERRs of *typical children*, the underlying distributions of responses are substantively different (Figure 7 in the main text).Supplementary Figures

## Figure S1 - Coefficient estimates of covariates associated with the egg reduction rate after treatment with praziquantel of a typical child infected with schistosomes.

Panels A-D depict fixed effects coefficients estimated from the classical conditional mixed models (panels A and C) and the analogous Bayesian models (panels B and D) fitted to the data on, *Schistosoma haematobium* (panels A and B) and *S. mansoni* (panels C and D) egg counts measured from children before and after treatment with praziquantel. The coefficient point estimates (black circles; posterior medians for the Bayesian models) indicate the multiplicative change (risk ratio, RR) in egg counts measured after treatment from a *typical child* in a particular covariate group compared to egg counts measured after treatment from *a typical child* in the reference group. Hence, a RR < 1 is associated with an *increased* egg reduction rate (ERR) and a RR > 1 is associated with a *decreased* ERR. Error bars depict 95% confidence intervals (CIs) for the classical estimates in panels A and C and analogous 95% credible intervals (also abbreviated to CI) in B and D. A covariate is deemed to exert a statistically significant effect only when its CI does not cross the vertical grey line at RR = 1. For example, a typical child infected with *S. mansoni* in Uganda or followed-up after 42 days is associated with a statistically significant *decrease* in ERR (RR > 1) compared to a typical child in Côte d’Ivoire or followed-up after 28 days.

# Supplementary Tables

## Table S1 - Summary of available datasets and results of applying exclusion criteria, adapted from Olliaro et al. [8]

| **Country** | **Participants (included)** | **Mean age (SD)^*^** | **QD protocol** | **PZQ regimen** | **FU days** | **Included** | **Reason for exclusion** | **Ref.** |
| --- | --- | --- | --- | --- | --- | --- | --- | --- |
| *Schistosoma haematobium* | | | | | | | | |
| Côte d’Ivoire | 18 (6^†^) | 5 (0) | 1 UF × 1 urine sample | 1×40 mg/kg | 21 | yes | NA | [9] |
| Niger | 377 (NA) | 9.1 (2.2) | 1 UF × 3 urine samples | 2×40 mg/kg | 42 | no | multiple doses; counts unavailable | [10] |
| Niger | 401 (NA) | 10.1 (2.3) | 1 UF × 3 urine samples | 2×40 mg/kg | 21 | no | multiple doses; counts unavailable | [10] |
| Côte d’Ivoire | 331 (NA) | 9.5 (2.6) | 1 UF × 1 urine sample | 2×40 mg/kg | 52 | no | multiple doses; FU > 6 weeks | [11] |
| Mali | 829 (NA) | 10.4 (2.3) | 1 UF × 1 urine sample | 1×40 mg/kg | 182.5 | no | FU > 6 weeks | [12] |
| Mali | 162 (NA) | 3.6 (1.2) | 1 UF × 1 urine sample | 1×40 mg/kg | 365 | no | FU > 6 weeks | [12] |
| Côte d’Ivoire | 87 (86^¶^) | 11.1 (1.9) | 1 UF × 2 urine samples | 1×40 mg/kg | 21 | yes | NA | [13] |
| Kenya | 95 (79^¶^) | 11.3 (3.1) | 1 UF × 4 urine samples | 1×40 mg/kg | 42 | yes | NA | [14] |
| *Schistosoma japonicum* | | | | | | | | |
| Philippines | 101 (NA) | 12.5 (2.0) | 2 KK × 2 stool samples | 1×40 mg/kg | 21 | no | only individuals with > 100 EPG enrolled | [15] |
| Philippines | 99 (NA) | 12.4 (2.0) | 2 KK × 2 stool samples | 1×60 mg/kg | 21 | no | only individuals with > 100 EPG enrolled | [15] |
| China | 100 (NA) | 46.1 (14.5) | 3 KK × 3 stool samples | 1×40 mg/kg | 90 | no | FU > 6 weeks | [16] |
| *Schistosoma mansoni* | | | | | | | |  |
| Côte d’Ivoire | 35 (35) | 3.8 (1.2) | 2 KK × 2 stool samples | 1×40 mg/kg | 21 | yes | NA | [9] |
| Uganda | 859 (503^¶^) | 4.2 (1.8) | 2 KK × 2 stool samples | 1×40 mg/kg | 21 | yes | NA | [17] |
| Niger | 217 (NA) | 9.48 (1.88) | 3 KK × 3 stool samples | 2×40 mg/kg | 42 | no | multiple doses; counts unavailable | [10] |
| Brazil | 96 (NA) | 15.2 (2.8) | 2 KK × 2 stool samples | 1×40 mg/kg | 21 | no | only individuals with > 100 EPG enrolled | [15] |
| Brazil | 94 (NA) | 14.9 (2.5) | 2 KK × 2 stool samples | 1×60 mg/kg | 21 | no | only individuals with > 100 EPG enrolled | [15] |
| Mauritania | 92 (NA) | 12.6 (2.0) | 2 KK × 2 stool samples | 1×40 mg/kg | 21 | no | only individuals with > 100 EPG enrolled | [15] |
| Mauritania | 93 (NA) | 12.6 (2.1) | 2 KK × 2 stool samples | 1×60 mg/kg | 21 | no | only individuals with > 100 EPG enrolled | [15] |
| Tanzania | 119 (NA) | 12.3 (1.8) | 2 KK × 2 stool samples | 1×40 mg/kg | 21 | no | only individuals with > 100 EPG enrolled | [15] |
| Tanzania | 125 (NA) | 12.7 (2.1) | 2 KK × 2 stool samples | 1×60 mg/kg | 21 | no | only individuals with > 100 EPG enrolled | [15] |
| Côte d’Ivoire | 177 (58^¶^) | 11.2 (4.1) | 1 KK × 3 stool samples | 1×40 mg/kg | 42 | yes | NA | [18] |
| Côte d’Ivoire | 49 (49) | 8.9 (2.4) | 2 KK × 2 stool samples | 1×40 mg/kg | 21 | yes | NA | [19] |
| Côte d’Ivoire | 85 (85) | 10.0 (1.42) | 1 KK x 4 stool samples | 1×40 mg/kg | 28 | yes | NA | [20] |
| Côte d’Ivoire | 270 (261^¶^) | 9.6 (2.1) | 1 KK x 5 stool samples | 1×60 mg/kg^∫^ | 28 | yes | NA | [21] |

Abbreviations: FU, follow-up; KK, Kato-Katz; NA, not applicable; PQZ, praziquantel; QD, quantitative diagnostic; SD, standard deviation; UF, urine filtration.

^*^ refers to included (analysed) data where applicable; ^†^ 12 preschool-aged schildren not included in analysis; ^¶^ participants excluded due to incomplete records;

^∫^ 2 doses of 30 mg/kg given 3 hours apart.

# Supplementary References

1. Lahiri SN. Resampling Methods for Dependent Data. New York: Springer; 2003
2. Diggle PJ: Analysis of Longitudinal Data**.** Oxford: Oxford University Press; 2002.
3. Elston DA, Moss R, Boulinier T, Arrowsmith C, Lambin X. Analysis of aggregation, a worked example: numbers of ticks on red grouse chicks. Parasitology 2001; 122:563-569.
4. Hadfield JD. MCMC methods for multi-response generalized linear mixed models: the MCMCglmm R package. J Stat Software 2010; 33:1-22.
5. Walker M, Churcher TS, Basáñez MG. Models for measuring anthelmintic drug efficacy for parasitologists. Trends Parasitol 2014; 30:528-537.

6. Gelman A, Carlin JB, Stern HS, Rubin DB. Bayesian Data Analysis. London: Chapman & Hall; 2004.

7. Hadfield JD. MCMCglmm Course Notes; 2014. [http://cran.r-project.org/web/packages/MCMCglmm/vignettes/CourseNotes.pdf \](http://cran.r-project.org/web/packages/MCMCglmm/vignettes/CourseNotes.pdf%20\)

8. Olliaro PL, Vaillant M, Diawara A, et al. Toward measuring *Schistosoma* response to praziquantel treatment with appropriate descriptors of egg excretion. PLoS Negl Trop Dis 2015; 9:e0003821.

9. Coulibaly JT, Fürst T, Silué KD, Knopp S, Hauri D, Ouattara M, et al. Intestinal parasitic infections in schoolchildren in different settings of Côte d’Ivoire: effect of diagnostic approach and implications for control. Parasit Vectors 2012; 5:135.

10. Garba A, Lamine MS, Barkire N, Djibo A, Sofo B, Gouvras AN, et al. Efficacy and safety of two closely spaced doses of praziquantel against *Schistosoma haematobium* and *S. mansoni* and re-infection patterns in school-aged children in Niger. Acta Trop 2013; 128:334-344.

11. N’Goran E, Gnaka H, Tanner M, Utzinger J. Efficacy and side-effects of two praziquantel treatments against *Schistosoma haematobium* infection, among schoolchildren from Côte d’Ivoire. Ann Trop Med Parasitol 2003; 97:37-51.

12. Landouré A, Dembélé R, Goita S, Kané M, Tuinsma M, Sacko M, et al. Significantly reduced intensity of infection but persistent prevalence of schistosomiasis in a highly endemic region in Mali after repeated treatment. PLoS Negl Trop Dis 2012; 6: e1774.

13. Stete K, Krauth SJ, Coulibaly JT, Knopp S, Hattendorf J, Müller I, et al. Dynamics of *Schistosoma haematobium* egg output and associated infection parameters following treatment with praziquantel in school-aged children. Parasit Vectors 2012; 5:298.

14. Olds GR, King C, Hewlett J, Olveda R, Wu G, Ouma J, et al. Double-blind placebo-controlled study of concurrent administration of albendazole and praziquantel in schoolchildren with schistosomiasis and geohelminths. J Infect Dis 1999; 179:996-1003.

15. Olliaro PL, Vaillant MT, Belizario VJ, Lwambo NJ, Ouldabdallahi M, Pieri OS, et al. A multicentre randomized controlled trial of the efficacy and safety of single-dose praziquantel at 40 mg/kg vs. 60 mg/kg for treating intestinal schistosomiasis in the Philippines, Mauritania, Tanzania and Brazil. PLoS Negl Trop Dis 2011; 5:e1165.

16. Xu J, Guo JG, Wu XH, Zeng XJ, Yang WP, Zheng J, et al. Efficacy and adverse effects of film coated praziquantel for treatment of schistosomiasis japonica. Zhonghua Yu Fang Yi Xue Za Zhi 2009; 43:718-722 (article in Chinese).

17. Sousa-Figueiredo JC, Betson M, Atuhaire A, Arinaitwe M, Navaratnam AM, Kabatereine NB, et al. Performance and safety of praziquantel for treatment of intestinal schistosomiasis in infants and preschool children. PLoS Negl Trop Dis 2012; 6:e1864.

18. Raso G, N’Goran EK, Toty A, Luginbühl A, Adjoua CA, Tian-Bi NT, et al. Efficacy and side effects of praziquantel against *Schistosoma mansoni* in a community of western Côte d’Ivoire. Trans R Soc Trop Med Hyg 2004; 98:18-27.

19. Scherrer AU, Sjöberg MK, Allangba A, Traoré M, Lohourignon LK, Tschannen AB, et al. Sequential analysis of helminth egg output in human stool samples following albendazole and praziquantel administration. Acta Trop 2009; 109: 226-231.

20. Utzinger J, Booth M, N’Goran EK, Müller I, Tanner M, Lengeler C. Relative contribution of day-to-day and intra-specimen variation in faecal egg counts of *Schistosoma mansoni* before and after treatment with praziquantel. Parasitology 2001; 122:537-544.

21. Utzinger J, N'Goran EK, N'dri A, Lengeler C, Tanner M. Efficacy of praziquantel against *Schistosoma mansoni* with particular consideration for intensity of infection. Trop Med Int Health 2000; 5:771-778.
